# Assessing Exposure to Atrazine and Its Metabolites Using Biomonitoring

**DOI:** 10.1289/ehp.10141

**Published:** 2007-07-18

**Authors:** Dana B. Barr, Parinya Panuwet, Johnny V. Nguyen, Simeon Udunka, Larry L. Needham

**Affiliations:** Division of Laboratory Sciences, National Center for Environmental Health, Centers for Disease Control and Prevention, Atlanta, Georgia, USA

**Keywords:** atrazine, chlorotriazines, environmental, exposure assessment

## Abstract

**Background:**

Atrazine (ATZ) is the second most abundantly applied pesticide in the United States. When we assessed exposure to ATZ by measuring its urinary mercapturic acid metabolite, general population data indicated that < 5% of the population was exposed to ATZ-related chemicals (limit of detection < 0.8 ng/mL).

**Objectives:**

The aim of our study was to determine if we were underestimating ATZ exposure by measuring its urinary mercapturic acid metabolite and if the urinary metabole profile changed with the exposure scenario.

**Methods:**

We conducted a small-scale study involving 24 persons classified as high- (*n* = 8), low(*n* = 5), and environmental- (*n* = 11) exposed to ATZ. Using online solid phase extraction high performance liquid chromatography–tandem mass spectrometry, we measured nine ATZ-related metabolites in urine that included dealkylated, hydroxylated, and mercapturic acid metabolites.

**Results:**

We found that the urinary metabolite profiles varied greatly among exposure scenarios and among persons within each exposure scenario. Although diaminochlorotriazine (DACT) appeared to be the predominant urinary metabolite detected in each exposure category, the variation in proportion of total ATZ metabolites among persons was consistently large, suggesting that one metabolite alone could not be measured as a surrogate for ATZ exposure.

**Conclusions:**

We have likely been underestimating population-based exposures by measuring only one urinary ATZ metabolite. Multiple urinary metabolites must be measured to accurately classify exposure to ATZ and its environmental degradates. Regardless, DACT and desethylatrazine appear to be the most important metabolites to measure to evaluate exposures to ATZ-related chemicals.

Atrazine (ATZ; 2-chloro-4-ethylamino-6-isopropylamino-*s*-triazine) is a systemic triazine herbicide that blocks photosynthesis in broadleaf weeds and some grassy weeds (Laws and Hayes 1991). ATZ is currently one of the two most widely used agricultural pesticides in the United States, although from 1985 to 2001, it was the most abundantly applied pesticide (Donaldson et al. 2002; Kiely et al. 2004). Approximately 64–80 million pounds of ATZ are applied annually in the United States for agricultural and residential purposes; this amount has remained relatively constant over the past few decades (Kiely et al. 2004).

ATZ has been used predominantly in the Midwest (Nelson et al. 2001). About three-fourths of all field corn and sorghum are treated with ATZ annually for weed control. Seventy percent of the ATZ applied to corn and sorghum is used as a preemergence herbicide, and 30% is applied postemergence. ATZ is also used for weed control in sugarcane and wheat fields. In addition to agricultural uses, ATZ is used in residential turf applications in the Southeast, including use on golf courses and sod farms to control weeds (Nelson et al. 2001).

ATZ and its degradates are the most commonly detected pesticide(s) in ground and surface waters (Aaronson et al. 1980; Appel and Hudak 2001; Barbash et al. 2001; Barnes and Kalita 2001; Battaglin et al. 2000; Bushway et al. 1992; Clark and Clay et al. 2000; Goolsby 2000; Gaynor et al. 2002). It has been the subject of multiple monitoring programs conducted by various industry and government agencies, especially the U.S. Geological Survey (USGS) (Barbash et al. 2001). The frequent detection of ATZ and its degradates in streams, rivers, groundwater, and reservoirs is related directly to its volume of use, its tendency to persist in soils because of its resistance to photolysis and hydrolysis, and its ability to travel with water systems (Nelson et al. 2001). In water systems, ATZ typically undergoes dealkylation to form desethylatrazine (DEA), desisopropylatrazine (DIA), and the terminal dealklylation product diaminochlorotriazine (DACT) (Nelson et al. 2001) (Figure 1). In plants, ATZ is absorbed by the root system and tends to form hydroxylated metabolites that cannot generally be removed by washing of the vegetable products. Studies reported to the U.S. Environmental Protection Agency (EPA) have suggested that in animals the degradation products that retain the chlorine have biological activity similar to that of atrazine, whereas the hydroxylated metabolites do not retain their biological activity (Nelson et al. 2001). The biological end points observed in animals have been primarily endocrine-mediated, for example, affecting hypothalamic control of pituitary–ovarian function by modulation of luteinizing hormone release (Cooper et al. 2000; Gojmerac et al. 2004; McMullin et al. 2004) and mammary tumor production (Eldridge et al. 1994, 1999; O’Connor et al. 2000; Ueda et al. 2005; Wetzel et al. 1994). ATZ has also been reported to modulate aromatase activity *in vivo* and *in vitro* (Crain et al. 1997; Hecker et al. 2004) potentially resulting in hermaphroditic amphiphibians (Hayes et al. 2002), although this health outcome has been debated (Gammon et al. 2005; Renner 2002).

Metabolism of ATZ and its degradation products is a complex process resulting in multiple potential metabolites (Figure 1). Many human and animal studies have evaluated ATZ metabolism and the conclusions are variable (Table 1). Some rat and human studies identified DACT as the primary metabolite of ATZ (Bakke et al. 1972; Catenacci et al. 1990, 1993). Other human studies identified atrazine mercapturate (AM) (Figure 1), a glutathione-derived metabolite, as the predominant metabolite (Buchholz et al. 1999; Lucas et al. 1993; Perry et al. 2000). In total, 8–12 ATZ metabolites have been identified or postulated (Bakke et al. 1972; Buchholz et al. 1999; Catenacci et al. 1990, 1993; Perry et al. 2000).

The latter human studies led us to focus our efforts mainly on measuring AM during biomontoring in the pilot phase of the Agricultural Health Study (ca. 1994; Alavanja et al. 1996). AM was frequently detected in the urine of farmer applicators, their spouses, and sometimes children, typically at the highest concentrations on the day after ATZ application (WJ Driskell, unpublished data, 1995). Subsequently, we began measuring AM to evaluate environmental exposures to ATZ; however, this approach limited our ability to detect total exposure to ATZ-related compounds. In the Centers for Disease Control and Prevention (CDC) *National Report on Human Exposure to Environmental Chemicals* (CDC 2001, 2003, 2005), AM, the only ATZ metabolite measured, was typically detected in < 5% of participants, which did not correspond with its widespread use and frequent detection in ground, surface, and municipal water systems. Similarly, other studies reported low frequencies of detection (e.g., < 3%) of AM (Adgate et al. 2001; Lioy et al. 2000; MacIntosh et al. 1999), even though one of these studies reported frequent detection of ATZ in homes (Lioy et al. 2000).

The objective of our study was to evaluate multiple metabolites of ATZ in persons exposed in occupational and environmental scenarios. We wanted to determine if measurement of one metabolite was sufficient to estimate ATZ exposure relative to that in other individuals or whether multiple chemicals must be measured to accurately assess exposure to ATZ.

## Experiment

A detailed description of the analytical method can be found in Panuwet et al. (in press). Briefly, we introduced 200 μL urine onto a dual online solid-phase extraction (reversed-phase phenyl–hexyl and strong cation exchange) system, which was operated by a novel switching mechanism. Analytes were preconcentrated using high-performance liquid chromatography on a guard column, and the remaining urine components were washed off, using 10% methanol in 0.1% formic acid, into a waste container. The valves were switched, and the analytes were backwashed onto a reversed-phase or strong anion exchange analytic column and separated using a linear gradient beginning with 10% methanol in 0.1% formic acid and ending with 100% methanol. Samples were analyzed using atmospheric pressure chemical ionization–tandem mass spectrometry with one precursor–product ion pair being used for quantification and two precursor–product ion pairs being used for confirmation (Table 2). Quantification was achieved using isotope dilution calibration, for which isotopically labeled standards were available. When they were not available, the most closely eluting labeled standard was used for quantification. Each analytical run consisted of a full eight-standard calibration set, three positive (i.e., fortified urine samples at three levels spanning calibration range) and two negative (i.e., blank) control samples, and up to 75 unknown samples. The limits of detection ranged from 0.1 to 1 ng/mL, with relative standard deviations typically < 12% over the calibration range.

Human samples were collected from residential turf applicators (i.e., commercial lawn care applicators), nonapplicators with low-level exposures (i.e., nonoccupationally exposed individuals with documented ATZ exposure based upon detectable levels of AM in their urine) and volunteers in Georgia with no known acute exposure to ATZ. All samples were collected as part of previous studies and were reanalyzed to determine total metabolites. All protocols were reviewed and approved by the CDC Institutional Review Board for ethical treatment of human research subjects. Samples were stored at −70°C until used and before analysis were thawed at room temperature and mixed thoroughly.

Simple statistics (mean, standard deviation, and ratios) were performed using Excel software (Microsoft, San Jose, CA). Because the number of samples tested was small, no significance testing was performed.

## Results

**Figure 2:**
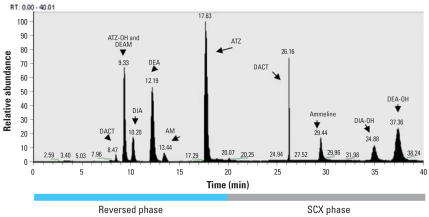
Mass chromatogram of metabolites measured. AM, atrazine mercapturate; ATZ, atrazine; ATZ-OH, hydroxyatrazine; DACT, diaminochlorotriazine; DEA, desethylatrazine; DEAM, desethylatrazine mercapturate; DEA-OH, hydroxydesethylatrazine; DIA, desisopropyl atrazine; DIA-OH, hydroxydesisopropylatrazine; SCX, strong cation exchange.

A mass chromatogram of a spiked urine sample is shown in Figure 2. Because the most polar analytes were not adequately retained on the reversed-phase column, a strong cation exchange column was used. DACT was partially retained on the reversed-phase column, and the remainder was retained on the strong cation exchange column resulting in two peaks for DACT. Both peaks were summed to calculate the total amount of DACT present. All peaks were resolved by either time or mass-to-charge ratio.

**Figure 3:**
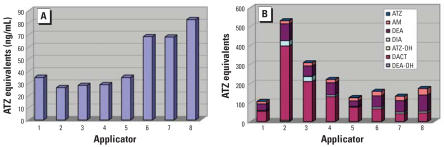
Metabolite profile of turf applicators using just AM and multiple metabolites. AM, atrazine mercapturate; ATZ, atrazine; ATZ-OH, hydroxyatrazine; DACT, diaminochlorotriazine; DEA, desethylatrazine; DEA-OH, hydroxydesethylatrazine; DIA, desisopropyl atrazine.

The metabolite profile of the higher exposure category of turf applicators is shown in Figure 3 (*n* = 8). The two graphs represent the exposure assessment using only AM and the exposure assessment using all metabolites. DACT was the most predominantly detected metabolite (mean = 51%), followed closely by DEA (mean = 31%). AM was detected on average in only 12% of the samples tested. The interperson variation (calculated as the relative standard deviation of the percentage of each metabolite percentage among persons) in urinary concentrations among these most detected analytes was between 33 and 51%. The interperson variability was much greater for the less frequently detected metabolites.

**Figure 4:**
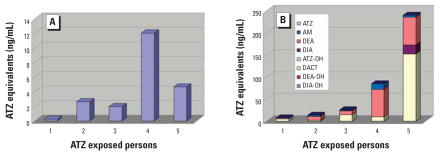
Metabolite profile of lower level exposures using AM and multiple metabolites. Abbreviations: AM, atrazine mercapturate; ATZ, atrazine; ATZ-OH, hydroxyatrazine; DACT, diaminochlorotriazine; DEA, desethylatrazine; DEA-OH, hydroxydesethylatrazine; DIA, desisopropyl atrazine; DIA-OH, hydroxydesisopropylatrazine.

The metabolite profiles for the lower-level exposure category (*n* = 5) are shown in Figure 4. On average, DEA (33%) and DACT (28%) were detected in about equal proportions, with only 6% detection of AM. Similar to the higher-level exposures, the interperson variation in their urinary concentrations was large.

For the environmental exposure category, DACT was by far the most predominantly detected metabolite (77%). DEA was detected the next most frequently (15%) and AM was detected in only 2% of the samples. Again, the interperson variability in metabolic profile concentrations was large.

## Discussion

The small amount of data that we present here clearly demonstrate that exposure to ATZ-related chemicals can be misrepresented by measurement of AM alone. However, it is important to note that the measurement of ATZ or AM in urine would be the only unequivocal indication that a person was exposed to ATZ and not an environmental degradate.

**Figure 5:**
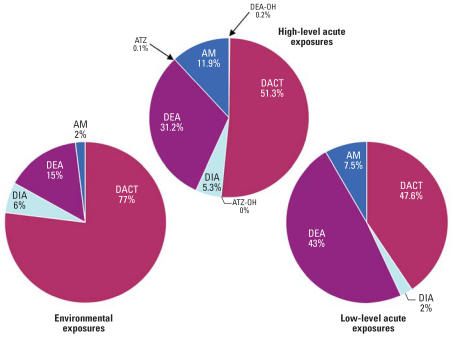
Average percent contribution of each metabolite to the total atrazine-related metabolite level for high, lower, and environmental exposures. Abbreviations: ATZ, atrazine; ATZ-OH, hydroxyatrazine; AM, atrazine mercapturate; DACT, diaminochlorotriazine; DEA, desethylatrazine; DEA-OH, hydroxydesethylatrazine; DIA, desisopropyl atrazine.

Also, the metabolite profiles differ dramatically based upon the exposure scenario (Figure 5). Occupational or lower-level acute exposures, perhaps after ATZ use on lawns, are probably more likely to be direct exposures to ATZ and lesser exposures to the degradation products. However, the environmental exposure scenario is quite different. In environmental exposures, persons likely are exposed through food or water, which would mean that dealkylation and hydrolysis products might make up a larger percentage of the exposure. Of course, exposure to the dealkylation products is still important because these chemicals remain biologically active. Thus, the presence of the chlorinated dealkylation products or their glutathione-mediated mercapturic acid metabolites would indicate exposure to a biologically active component. Presence of the hydroxylated metabolites may indicate exposure to the hydroxylated products themselves or to their chlorinated counterparts.

Our data demonstrate that we likely will need to measure most or all metabolites of ATZ to accurately assess ATZ-related exposures. However, further evaluation is necessary because of our small sample size and because the possible presence of glucuronide metabolites was not considered. In the future, we will explore further the role of hydroxyl metabolites in the metabolite profiles by evaluating glucuronide-hydrolyzed urine. Also, we need to include the mercapturates of the dealkylation products in our methodology to glean the full picture of ATZ metabolism. Futhermore, we will use this method to evaluate larger populations with high-level occupational exposures (e.g., manufacturers, farmers) and background exposures in the general U.S. population.

Although we found detectable concentrations of ATZ metabolites in most of the urine samples tested, we are uncertain what, if any, health effects result from these levels of exposure. In general, animal dosing studies that have investigated health effects (Ashby et al. 2002; Cooper et al. 1996, 2000) have used doses much larger than those to which we could assume (from back calculation) participants were exposed in our study. Further studies evaluating health outcomes at typical human exposure levels are warranted.

## Conclusions

We have clearly been underestimating exposure to ATZ-related metabolites in the U.S. population and in other selected studies. Our newer data are more in line with exposures we might expect to see based upon ATZ use and environmental persistence. It is likely that multiple metabolites must be measured to accurately classify exposure categories. Although DACT appeared to be the predominant metabolite detected in each exposure category, the interperson variations in its concentrations were consistently about 30%. Regardless, DACT and DEA appear to be the most important metabolites to measure to evaluate exposures to ATZ-related chemicals.

Clearly, exposure to ATZ or its degradates appears more pervasive than previously believed; however, more data are needed to confirm this observation. Where measures exist to mitigate or lessen exposures to biologically active ATZ degradates such as the use of high efficiency filters in municipal water systems or other mitigation strategies for pond and surface waters (Acosta et al. 2004; Agdi et al. 2000), they should be used to ensure the best protection of public health.

## Correction

The percent contribution for “Low-level acute exposures” in Figure 5 was changed from that in the original manuscript published online.

## Figures and Tables

**Figure 1:**
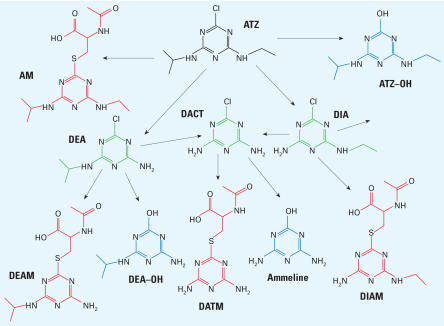
. Proposed metabolism of ATZ. ATZ is shown in black; dealkylated metabolites are shown in green; hydroxylated metabolites are shown in blue; and glutathione-derived mercapturic acid metabolites are shown in red. Abbreviations: ATZ, atrazine; ATZ-OH, hydroxyatrazine; AM, atrazine mercapturate; DACT, diaminochlorotriazine; DATM, diaminotriazine mercapturate; DEA, desethylatrazine; DEAM, desethylatrazine mercapturate; DEA-OH, hydroxydesethylatrazine; DIA, desisopropyl atrazine; DIAM, desisopropylatrazine mercapturate.

**Table 1:** Summary of animal and human metabolite studies.^a^

Study	Model	DACT	DIA	DEA	AM	ATZ	DAtM	ATZ-OH	Ammeline
Novartis^b^	Rat	1	Minor	Minor	ND	ND	1	ND	ND
Bakke (Bakke et al. 1972)	Rat	ND	1	1	ND	ND	ND	1	1
DE Bradway^b^	Rat	1	1	1	NA	NA	NA	1	1
LL Erickson^b^	Swine	NA	NA	1	NA	NA	NA	NA	NA
Novartis^b^	Monkey	1	4	2	3	ND	After iv	ND	ND
Catenacci (Catenacci et al. 1993)	Human	1	Minor	Minor	NA	Minor	NA	NA	NA
Lucas (Lucas et al. 1993)	Human (D)	Minor	Minor	Minor	1	NA	NA	NA	NA
Buchholz (Buchholz et al. 1999)	Human (D)	2?	Minor	NA	1	NA	2?	NA	NA
Perry (Perry et al. 2000)	Human	NA	NA	2	1	NA	NA	NA	NA
Catenacci (Catenacci et al. 2002)	Human	1	Minor	2	NA	Minor	NA	NA	NA

Abbreviations: AM, atrazine mercapturate; ATZ, atrazine; ATZ-OH, hydroxyatrazine; DACT, diaminochlorotriazine; DATM, diaminotriazine mercapturate; DEA, desethylatrazine; DIA, desisopropyl atrazine; ND, not detected; NA, not measured or applicable to the study; ?, metabolite with tentative identification; (D), dermal exposure. “After iv” indicates that metabolite was seen only after iv administration of atrazine.

a 1, a major metabolite; 2, a less abundant metabolite; minor, minor metabolite identified.

b Information obtained from documentation of internal studies conducted at Novartis. Information was kindly supplied by Novartis upon request by CDC.

**Table 2:** Precursor–product ion pairs for tandem mass spectrometry of each analyte.^a^

		Productions		
Compound	Precursor ion [M+H]	Q	C1	C2
ATZ-OH	198	86	156	69
DACT	146	79	68	62
DEAM	315	185	144	102
DIA	174	68	132	104
DEA	188	146	104	110
AM	343	214	102	172
ATZ	216	174	104	68

Abbreviations: AM, atrazine mercapturate; ATZ-OH, hydroxyatrazine; ATZ, atrazine; C1, confirmation ion 1; C2, confirmation ion 2; [M+H], pseudomolecular ion derived from atmospheric pressure chemical ionization; DACT, diaminochlorotriazine; DATM, diaminotriazine mercapturate; DEA, desethylatrazine; DEAM, desethylatrazine mercapturate; DIA, desisopropyl atrazine; Q, quantification ion.

a More details on the method are found in Panuwet et al. (in press).

## References

[b1-ehp0115-001474] Aaronson MJ, Kirby KW, Tessari JD (1980). Identification and confirmation of atrazine in pond water. Bull Environ Contam Toxicol.

[b2-ehp0115-001474] Acosta EJ, Steffensen MB, Tichy SE, Simanek EE (2004). Removal of atrazine from water using covalent sequestration. J Agric Food Chem.

[b3-ehp0115-001474] Adgate JL, Barr DB, Clayton CA, Eberly LE, Freeman NC, Lioy PJ (2001). Measurement of children’s exposure to pesticides: analysis of urinary metabolite levels in a probability-based sample. Environ Health Perspect.

[b4-ehp0115-001474] Agdi K, Bouaid A, Esteban AM, Hernando PF, Azmani A, Camara C (2000). Removal of atrazine and four organophosphorus pesticides from environmental waters by diatomaceous earth-remediation method. J Environ Monit.

[b5-ehp0115-001474] Alavanja MC, Sandler DP, McMaster SB, Zahm SH, McDonnell CJ, Lynch CF (1996). The Agricultural Health Study. Environ Health Perspect.

[b6-ehp0115-001474] Appel PL, Hudak PF (2001). Automated sampling of stormwater runoff in an urban watershed, north-central Texas. J Environ Sci Health A Tox Hazard Subst Environ Eng.

[b7-ehp0115-001474] Ashby J, Tinwell H, Stevens J, Pastoor T, Breckenridge CB (2002). The effects of atrazine on the sexual maturation of female rats. Regul Toxicol Pharmacol.

[b8-ehp0115-001474] Bakke JE, Larson JD, Price CE (1972). Metabolism of atrazine and 2-hydroxyatrazine by the rat. J Agric Food Chem.

[b9-ehp0115-001474] Barbash JE, Thelin GP, Kolpin DW, Gilliom RJ (2001). Major herbicides in ground water: results from the National Water-Quality Assessment. J Environ Qual.

[b10-ehp0115-001474] Barnes PL, Kalita PK (2001). Watershed monitoring to address contamination source issues and remediation of the contaminant impairments. Water Sci Technol.

[b11-ehp0115-001474] Battaglin WA, Furlong ET, Burkhardt MR, Peter CJ (2000). Occurrence of sulfonylurea, sulfonamide, imidazolinone, and other herbicides in rivers, reservoirs and ground water in the Midwestern United States, 1998. Sci Total Environ.

[b12-ehp0115-001474] Buchholz BA, Fultz E, Haack KW, Vogel JS, Gilman SD, Gee SJ (1999). HPLC-accelerator MS measurement of atrazine metabolites in human urine after dermal exposure. Anal Chem.

[b13-ehp0115-001474] Bushway RJ, Hurst HL, Perkins LB, Tian L, Cabanillas CG, Young BE (1992). Atrazine, alachlor, and carbofuran contamination of well water in central Maine. Bull Environ Contam Toxicol.

[b14-ehp0115-001474] Catenacci G, Barbieri F, Bersani M, Ferioli A, Cottica D, Maroni M (1993). Biological monitoring of human exposure to atrazine. Toxicol Lett.

[b15-ehp0115-001474] Catenacci G, Colli G, Verni P, Barisano A (2002). Environmental and biologic monitoring of atrazine exposure at a formulating plant. G Ital Med Lav Ergon.

[b16-ehp0115-001474] Catenacci G, Maroni M, Cottica D, Pozzoli L (1990). Assessment of human exposure to atrazine through the determination of free atrazine in urine. Bull Environ Contam Toxicol.

[b17-ehp0115-001474] CDC (2001). National Report on Human Exposure to Environmental Chemicals. http://www.cdc.gov/exposurereport.

[b18-ehp0115-001474] CDC (2003). Second National Report on Human Exposure to Environmental Chemicals. http://www.cdc.gov/exposurereport.

[b19-ehp0115-001474] CDC (2005). Third National Report on Human Exposure to Environmental Chemicals. http://www.cdc.gov/exposurereport.

[b20-ehp0115-001474] Clark GM, Goolsby DA (2000). Occurrence and load of selected herbicides and metabolites in the lower Mississippi River. Sci Total Environ.

[b21-ehp0115-001474] Clay SA, Dowdy RH, Lamb JA, Anderson JL, Lowery B, Knighton RE (2000). Herbicide movement and dissipation at four Midwestern sites. J Environ Sci Health B.

[b22-ehp0115-001474] Cooper RL, Stoker TE, Goldman JM, Parrish MB, Tyrey L (1996). Effect of atrazine on ovarian function in the rat. Reprod Toxicol.

[b23-ehp0115-001474] Cooper RL, Stoker TE, Tyrey L, Goldman JM, McElroy WK (2000). Atrazine disrupts the hypothalamic control of pituitary-ovarian function. Toxicol Sci.

[b24-ehp0115-001474] Crain DA, Guillette LJ, Rooney AA, Pickford DB (1997). Alterations in steroidogenesis in alligators *(Alligator mississippiensis*) exposed naturally and experimentally to environmental contaminants. Environ Health Perspect.

[b25-ehp0115-001474] Donaldson D, Kiely T, Grube A (2002). 1998 and 1999 Market Estimates. Pesticides Industry Sales and Usage Report.

[b26-ehp0115-001474] Eldridge JC, Tennant MK, Wetzel LT, Breckenridge CB, Stevens JT (1994). Factors affecting mammary tumor incidence in chlorotriazine-treated female rats: hormonal properties, dosage, and animal strain. Environ Health Perspect.

[b27-ehp0115-001474] Eldridge JC, Wetzel LT, Stevens JT, Simpkins JW (1999). The mammary tumor response in triazine-treated female rats: a threshold-mediated interaction with strain and species-specific reproductive senescence. Steroids.

[b28-ehp0115-001474] Gammon DW, Aldous CN, Carr WC, Sanborn JR, Pfeifer KF (2005). A risk assessment of atrazine use in California: human health and ecological aspects. Pest Manag Sci.

[b29-ehp0115-001474] Gaynor JD, Tan CS, Drury CF, Welacky TW, Ng HY, Reynolds WD (2002). Runoff and drainage losses of atrazine, metribuzin, and metolachlor in three water management systems. J Environ Qual.

[b30-ehp0115-001474] Gojmerac T, Pleadin J, Zuric M, Rajkovic-Janje R, Korsic M (2004). Serum luteinizing hormone response to administration of gonadotropin-releasing hormone to atrazine-treated gilts. Vet Hum Toxicol.

[b31-ehp0115-001474] Hayes TB, Collins A, Lee M, Mendoza M, Noriega N, Stuart AA (2002). Hermaphroditic, demasculinized frogs after exposure to the herbicide atrazine at low ecologically relevant doses. Proc Natl Acad Sci USA.

[b32-ehp0115-001474] Hecker M, Giesy JP, Jones PD, Jooste AM, Carr JA, Solomon KR (2004). Plasma sex steroid concentrations and gonadal aromatase activities in African clawed frogs (*Xenopus laevis*) from South Africa. Environ Toxicol Chem.

[b33-ehp0115-001474] Kiely T, Donaldson D, Grube A (2004). Pesticide industry sales and usage: 2000 and 2001 market estimates.

[b34-ehp0115-001474] Laws ER, Hayes WJ (1991). Handbook of Pesticide Toxicology.

[b35-ehp0115-001474] Lioy PJ, Edwards RD, Freeman N, Gurunathan S, Pellizzari E, Adgate JL (2000). House dust levels of selected insecticides and a herbicide measured by the EL and LWW samplers and comparisons to hand rinses and urine metabolites. J Expo Anal Environ Epidemiol.

[b36-ehp0115-001474] Lucas AD, Jones AD, Goodrow MH, Saiz SG, Blewett C, Seiber JN (1993). Determination of atrazine metabolites in human urine: development of a biomarker of exposure. Chem Res Toxicol.

[b37-ehp0115-001474] MacIntosh DL, Needham LL, Hammerstrom KA, Ryan PB (1999). A longitudinal investigation of selected pesticide metabolites in urine. J Expo Anal Environ Epidemiol.

[b38-ehp0115-001474] McMullin TS, Andersen ME, Nagahara A, Lund TD, Pak T, Handa RJ (2004). Evidence that atrazine and diaminochlorotriazine inhibit the estrogen/progesterone induced surge of luteinizing hormone in female Sprague-Dawley rats without changing estrogen receptor action. Toxicol Sci.

[b39-ehp0115-001474] Nelson H, Lin J, Frankenberry M (2001). Drinking Water Exposure Assessment for Atrazine and Various Chloro-triazine and Hydroxy-triazine Degradates.

[b40-ehp0115-001474] O’Connor JC, Plowchalk DR, Van Pelt CS, Davis LG, Cook JC (2000). Role of prolactin in chloro-*S*-triazine rat mammary tumorigenesis. Drug Chem Toxicol.

[b41-ehp0115-001474] Perry M, Christiani D, Dagenhart D, Tortorelli J, Singzoni B (2000). Urinary biomarkers of atrazine exposure among farm pesticide applicators. Ann Epidemiol.

[b42-ehp0115-001474] Panuwet P, Nguyen JV, Udunka S, Kuklenyik P, Needham LL, Barr DB Quantitation of urinary atrazine and its metabolites by on-line solid phase extraction-high performance liquid chromatography-tandem mass spectrometry. J Expo Science Environ Epidemiol.

[b43-ehp0115-001474] Renner R (2002). Amphibian declines. Conflict brewing over herbicide’s link to frog deformities. Science.

[b44-ehp0115-001474] Ueda M, Imai T, Takizawa T, Onodera H, Mitsumori K, Matsui T (2005). Possible enhancing effects of atrazine on growth of 7,12-dimethylbenz(a)anthracene-induced mammary tumors in ovariectomized Sprague-Dawley rats. Cancer Sci.

[b45-ehp0115-001474] Wetzel LT, Luempert LG, Breckenridge CB, Tisdel MO, Stevens JT, Thakur AK (1994). Chronic effects of atrazine on estrus and mammary tumor formation in female Sprague-Dawley and Fischer 344 rats. J Toxicol Environ Health.

